# Identification of Novel *ARSA* Mutations in Chinese Patients with Metachromatic Leukodystrophy

**DOI:** 10.1155/2018/2361068

**Published:** 2018-07-03

**Authors:** Li Chen, Huifang Yan, Binbin Cao, Ye Wu, Qiang Gu, Jiangxi Xiao, Yanling Yang, Huixia Yang, Zhen Shi, Zhixian Yang, Hong Pan, Xingzhi Chang, Junya Chen, Yu Sun, Yuehua Zhang, Xiru Wu, Yuwu Jiang, Jingmin Wang

**Affiliations:** ^1^Department of Pediatrics, Peking University First Hospital, Beijing 100034, China; ^2^Department of Pediatric Cardiology, Beijing Anzhen Hospital, Capital Medical University, Beijing 100029, China; ^3^Department of Radiology, Peking University First Hospital, Beijing 100034, China; ^4^Department of Obstetrics and Gynecology, Peking University First Hospital, Beijing 100034, China; ^5^Department of Central Laboratory, Peking University First Hospital, Beijing 100034, China

## Abstract

**Objective:**

Metachromatic leukodystrophy (MLD) is an inherited disease caused by a deficiency of the enzyme arylsulfatase A (ARSA) that leads to severe physiologic and developmental problems. Our study is aimed at elucidating the clinical and genetic characteristics of Chinese MLD patients.

**Methods:**

Clinical data of 21 MLD patients was collected. All coding exons of *ARSA* and their flanking intronic sequences were amplified by polymerase chain reaction and subjected to direct sequencing.

**Results:**

All 21 patients were diagnosed with MLD clinically and genetically, out of which 17 patients were late infantile and 4 were juvenile types. A total of 34 *ARSA* mutations, including 28 novel mutations (22 missense, 1 splicing, 1 nonsense, 3 small insertions, and 1 small deletion mutation) and 6 known mutations (5 missense and 1 small insertion mutation), were identified. Prenatal diagnosis was performed for four pedigrees. One fetus was a patient, two fetuses were carriers, and two were wild type.

**Conclusions:**

The present study discovered 28 novel *ARSA* mutations and widely expanded the mutation spectrum of *ARSA*. Four successful prenatal diagnoses provided critical information for MLD families.

## 1. Introduction

Metachromatic leukodystrophy (MLD) is a genetic disorder caused by the deficiency of the enzyme arylsulfatase A (ARSA) in lipid metabolism [1–3]. It is estimated that the overall incidence of autosomal recessive MLD is 1 : 40,000–1 : 100,000 [1]. The classical symptoms are presented mainly as progressive physical and mental deterioration, clumsiness, frequent falls, toe walking, slurred speech, weakness, hypotonia, and seizures. MLD patients could be classified into three subtypes, including late infantile, juvenile, and adult forms based on the age of the first symptom onset [1]. The late infantile form usually manifests in the second year of life. The juvenile form, with an onset between 4 and 15 years, is further subdivided into early juvenile and late juvenile depending on whether the onset is before or after 6 years of age. The adult form has an onset older than 15 years of age [1, 4–6]. Magnetic resonance imaging and biochemical assays are commonly used for diagnosis [7–9]. Measuring ARSA enzymatic activity in leukocytes from whole blood is the standard biochemical procedure [10]. However, diagnosis of MLD would be based on *ARSA* diagnosis. *ARSA* is located on chromosome 22q13 containing eight exons and is transcribed into three mRNA species with a total length of 3.2 kb. Currently, the Human Gene Mutation Database (http://www.hgmd.cf.ac.uk/ac/gene.php?gene=ARSA) has reported a total of 217 *ARSA* mutations. Among them are c.459+1G>A and c.1277C>T (pPro426Leu), which occur more frequently in the European population with over 200 reported mutations in MLD patients [10]. The former causes a splice donor site mutation, which occurs frequently in late infantile patients, whereas the latter appears in adult or juvenile patients [10]. In addition to pathogenic mutations, an ARSA pseudodeficiency allele, such as c.1049A>G, leads to lower ARSA activity, which results in a partial mistargeting of the enzyme [11]. The aim of this study was to elucidate the clinical and genetic characteristics of Chinese MLD patients. Additionally, for MLD pedigrees diagnosed genetically, we performed prenatal diagnosis.

## 2. Materials and Methods

### 2.1. Patients

Twenty-one MLD patients (Pt1-Pt21) from twenty-one unrelated families were enrolled in this study at the Department of Pediatrics at Peking University First Hospital. All patients were clinically diagnosed with MLD based on the manifestation, classical MRI feature, and ARSA enzyme deficiency in leukocytes. Out of 21 patients, thirteen patients were male (61.90%) and eight patients were female (38.10%). The median age was 32 months (minimum 20 months, maximum 11 years old). Clinical characteristics of patients were collected, such as onset age, symptoms, neurological findings, and brain MRI findings. Our study was conducted with the patients' understanding and consent. The study was approved by the Ethics Committee of Peking University First Hospital, and informed consent was obtained from the patients' legal guardians.

### 2.2. Biochemical Studies

Patient studies were performed using low-temperature assays of arylsulfatase A activity determination in leukocytes [12], and the patients demonstrated low ARSA activity (<10% normal range).

### 2.3. Mutation Analysis

Genomic DNA was extracted from peripheral blood leukocytes of patients and their parents according to the standard protocols of the QIAamp DNA blood mini kit (Qiagene Inc.). Using reported PCR primers and annealing temperatures [13], *ARSA* was amplified and the PCR products were purified by a DNA purification kit. PCR conditions were as follows: 100 ng of patient DNA was amplified in 10 *μ*L of buffer containing 0.5 *μ*L of 5 mmol/L dNTP, 50 ng of each primer, 5 *μ*L of 2x GC buffer (Takara, Dalian, China), and 1 *μ*L of Taq DNA polymerase (Tiangen, Beijing, China). Then, the PCR products were subject to an automated sequencer. The samples collected before 2013 were sequenced by the Sanger sequencing method, whereas samples collected after 2013 were sequenced by PANO-seq. The putative mutations were confirmed using duplicate PCR products or digested PCR products. The effect of the amino acid changes in ARSA was predicted by the web server PolyPhen-2 with the HumDiv model (http://genetics.bwh.harvard.edu/pph2/).

For prenatal diagnosis, chorionic villus biopsy (CVS) or amniocentesis was performed for fetuses in four MLD families (Pt5, Pt7, Pt15, and Pt18) to obtain samples. A Promega Wizard® Genomic DNA Purification Kit (A1120) was used to extract the genomic DNA.

### 2.4. RNA Isolation from Peripheral Blood Cells and Detection of *ARSA* mRNA

Total RNA was prepared from blood cells using the TRIzol reagent (Invitrogen, Waltham, MA, USA) according to the manufacturer's instructions. Five hundred nanograms of RNA was reverse transcribed using oligo d(T)_20_, and the complementary DNA was added to the quantitative real-time PCR assays.

## 3. Results

### 3.1. Clinical Finding

**Figure 1:**
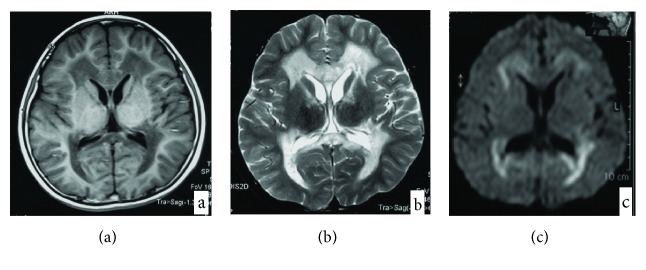
Magnetic resonance imaging (MRI) shows symmetrical deep lesions located in periventricular white matter, which was low signal in T1WI (a), high signal in T2WI (b), and high signal in DWI (c).

Clinical data were collected and are shown in Table 1. Ages at onset ranged from 6 months to 5 years old, with a median age of 25 months. Out of 21 patients, the main complaints from the 10 patients were motor regression, 4 with both motor and intelligence regression, 6 showing development retardation, and 1 with abnormal walking posture. Symmetrical abnormalities in deep white matter (WM) (Figure 1) were observed in MRI images in all 21 patients. ARSA activity was measured in 14 patients, all of which were lower than 10% of the normal range. A Babinski sign was positive in all patients except Pt1, Pt5, and Pt8.

All patients were clinically diagnosed with MLD according to the clinical manifestations described above (Table 1). Out of 21 patients, 17 late infantile-type and 4 juvenile-type patients were diagnosed based on their onset age of younger than 30 mon and older than 30 mon, respectively.

### 3.2. Genetic Findings

Thirty-four mutations, including 28 novel and 6 known mutations, were found in *ARSA* (Table 2). The 28 novel mutations included 22 missense mutations, 3 small insertions, 1 splicing mutation, 1 small deletion, and 1 nonsense mutation. Among these novel mutations, the mutation c.1130_1132delTCT caused a three base pair deletion in *ARSA*, while the mutation c.954G>A, pTrp318Term produced a premature termination code, the mutations c.1344_1345insCC, c.302_303insG, and c.1428_1429insC caused one or two base pair insertion in *ARSA*, and the mutations c.1108-20A>G, c.465G>A (p.Lys125ProfsX17), and IVS3+2T>C led to splicing and amino acid changes in the protein. Mutations c.1172T>G (p.Val391Gly), c.827C>T (p.Thr276Met), c.925G>A (pGlu309Lys), and c.1130_1132delTCT were all detected in 2 patients, respectively. After predicting the effect of the amino acid change in ARSA by the web server PolyPhen-2 with the HumDiv model, we found that 28 mutations were probably damaging to the protein activity with a high score and specificity. Six known mutations have been reported, including c.917C>T, c.827C>T, c.179_180insCA, c.257G>A, c.925G>A, and c.302G>T.

Out of 21 patients, 2 (Pt2 and Pt3) harbored homozygous mutations in *ARSA* and 19 had compound heterozygous mutations in *ARSA*. Two patients had homozygous mutations of *ARSA* that were inherited from their parents. All 19 patients with compound heterozygous mutations of *ARSA* had mutations that were inherited from their parents, except for Pt14 and Pt19. The *ARSA* c.465G>A (p.Lys125ProfsX17) mutation in Pt14 was inherited from the father with a heterozygous variation on one allele and the mother with a wild-type mutation on the other allele. Pt19 has spontaneous splicing mutation IVS3+2T>C.

In Pt5, Pt7, Pt15, and Pt18 MLD family genetic diagnoses, prenatal diagnosis was performed for fetuses after the second or third pregnancy (Table 3). All samples were tested by a short tandem repeat (STR) linkage analysis to exclude contamination from the mother's tissue. The results showed that the fetuses had wild-type *ARSA* in the Pt5 (c.917C>T and c.827C>T) and Pt15 (c.44G>T and c.610C>G) cases. While one fetus in the Pt7 MLD family had a compound heterozygous mutation (c.1130_1132delTCT and c.1238A>G), another fetus in Pt7 carried one heterozygous variation (c.1238A>G (p.Asp413Gly)). The compound heterozygous mutations c.302_303insG and c.1428_1429insC were detected in fetuses of Pt18's mother.

## 4. Discussion

MLD is a kind of lysosomal storage disorder due to the deficiency of the ARSA enzyme, which is involved in the metabolism of membrane sulfatides into galactosylceramide. Progressive demyelination and dysfunction of the peripheral and central nervous systems is the symptom of this disease as the undegraded sulfatides require time to accumulate in oligodendrocytes and Schwann cells. In this study, 21 patients presented typical clinical symptoms, including motor regression, motor and intelligence regression, development retardation, and abnormal walking posture. They all had typical brain MRI findings. Fourteen patients had lower than 10% normal activity of the ARSA outcome. According to the above clinical manifestations described, all patients were clinically diagnosed as MLD. All the patients' ages at onset ranged from 6 months to 5 years old. Out of 21 patients, 17 patients of younger than 24 mon were diagnosed as the late infantile type and the remaining 4 patients as juvenile type.

*ARSA*, the disease-causing gene of MLD, is located on chromosome 22q13, has a total length of 3.2 kb, contains eight exons, and is transcribed into three mRNA species. To date, the Human Gene Mutation Database (http://www.hgmd.cf.ac.uk/ac/gene.php?gene=ARSA) has reported a total of 217 *ARSA* mutations, including 161 missense mutations, 14 splicing-site mutations, 20 small deletions, 12 small insertions, 4 small indels, 2 gross deletions, 2 complex rearrangements, 1 gross insertion, and 1 regulatory mutation. Here, 34 *ARSA* mutations were identified, which included 28 novel and 6 known mutations [4–9, 14]. Because most mutations in our study are novel mutations, the diagnosis of MLD should be confirmed not only by low ARSA activity but also by increased sulfatiduria. In our study, 28 novel mutations, including 22 missense mutations, 3 small insertions, 1 splicing mutation, 1 small deletion, and 1 nonsense mutation, and 6 reported mutations (c.917C>T, c.827C>T, c.179_180insCA, c.257G>A, c.925G>A, and c.302G>T) were found [4–9, 14].

For the 28 novel mutations, we predicted the effect of amino acid changes of the 22 missense mutations in ARSA by the web server PolyPhen-2 with the HumDiv model (Table 4). The scores of mutations, including 251C>A (pPro84Gln), 427T>C (p.Phe143Leu), 640G>A (p.Ala214Thr), and 754T>C (p.Ser252Pro), were 0.991–0.999. Therefore, we predicted that they could cause damage to the function of the ARSA protein. The mutational sensitivity of 427T>C (p.Phe143Leu), 640G>A (p.Ala214Thr), and 754T>C (p.Ser252Pro) was 0.14, 0.69, and 0.55, respectively. All the scores of the other missense mutations were 1, with a sensitivity of 0 and specificity of 1. They included the mutation of the terminal C domain. Therefore, we predicted that they could destroy the function of the ARSA protein. The mutation c.1130_1132delTCT caused a three base pair deletion in *ARSA*, and the mutation c.954G>A, pTrp318Term produced a premature termination codon, while the mutations c.1344_1345insCC, c.302_303insG, and c.1428_1429insC inserted one or two base pairs in *ARSA*. We predicted that the mutations c.1108-20A>G, c.465G>A (p.Lys125ProfsX17), and IVS3+2T>C resulted in splicing, which could cause damage to the function of ARSA protein. Based on this analysis, the 28 mutations are probably damaging to protein activity. All 6 known mutations, including c.917C>T, c.827C>T, c.179_180insCA, c.257G>A, c.925G>A, and c.302G>T, could destroy the function of the ARSA protein and were considered disease-causing mutations.

**Figure 2:**
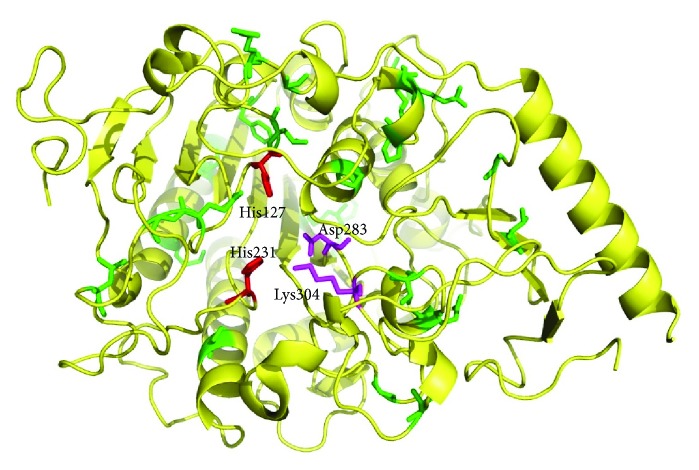
The illustration of *ARSA* 3D structure. Red and purple colors indicate the key residues to form reaction pocket. Green colors indicate the missense mutations in this study.

Through further analysis, we found that mutations c.251C>A, c.1049T>A, c.244C>T, c.296G>T c.911A>T, c.1238A>G, and c.925G>A lead to changes of the chemical properties in residues. The crystal structure of ARSA and the substrate also indicated that p.Lys304 is one of the key residues that interacted with the ester oxygen atom (O_4_) of the substrate in the reaction pocket [15] (Figure 2). Other mutations, which do not change the chemical properties of the residues, could also be important if they were localized near the reaction pocket. In human ARSA, lysine 123, lysine 302, serine 150, and histidine 229 were identified as the key residues that form the reaction pocket and interact with the substrate [15]. Here, the mutation p.Thr306Met near p.Lys302 in Pt4 and Pt5 resulted in the loss of enzyme activity in vitro. The mutation p.Asp283Glu in Pt9, which also does not change the chemical properties of the residue, also leads to MLD in previous studies. It suggests that p.Asp283Glu results in a loss of the interaction between ARSA and the substrate.

Unlike the reported studies in the European population, no patients were detected with mutations c.459+1G>A and c.1277C>T (pPro426Leu), which occurred with high frequency, according to the publication. The known mutation c.917C>T (p.Thr306Met) was first reported in Europe [16]. Mutation c.827C>T (PThr276Met) was found in a high frequency of MLD patients of Lebanese or Arabic descent with a high degree of consanguinity and a common ethnic origin [17]. In contrast to these studies, no highly frequent mutation was found in the current study of the Chinese population.

In 21 MLD patients, 2 (Pt2 and Pt3) had homozygous and 19 had compound heterozygous mutations of *ARSA*. They were all consistent with autosomal recessive inheritance. Two patients had homozygous mutations of *ARSA* that were inherited from their parents. All 19 patients with compound heterozygous mutations of *ARSA* had mutations that were inherited from their parents, except Pt14 and Pt19. The mutation of *ARSA* (c.465G>A (p.Lys125ProfsX17)) in Pt14 was inherited from the father with a heterozygous variation on one allele and the mother with a wild-type allele. Pt19 has the spontaneous splicing mutation IVS3+2T>C.

**Figure 3:**
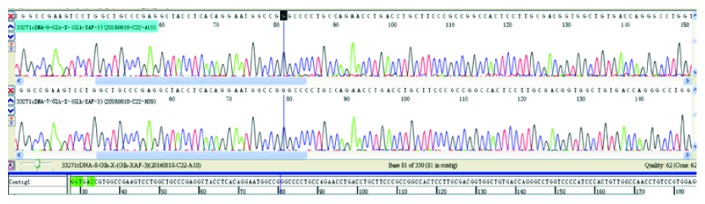
*ARSA* RNA-cDNA of Pt19.

**Figure 4:**
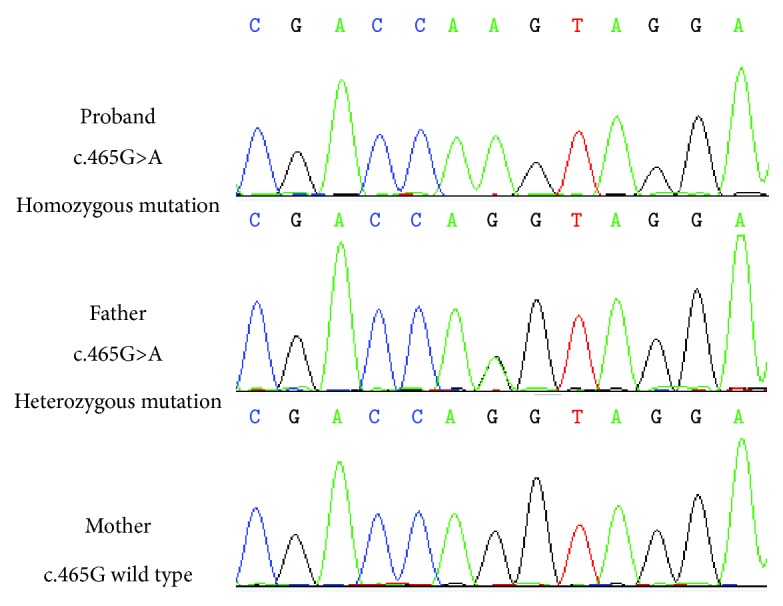
The *ARSA* mutation of Pt14, his father, and mother.

**Figure 5:**
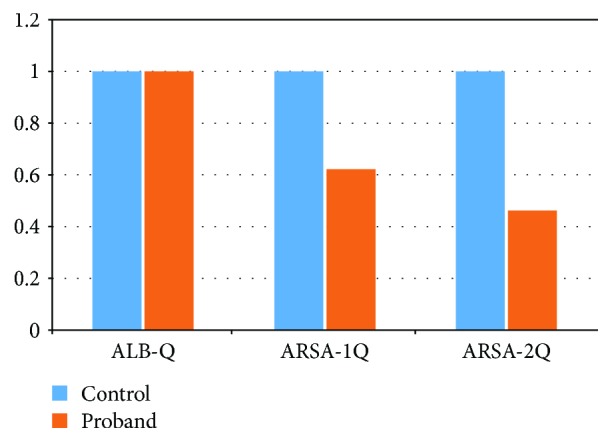
The fluorescent quantitation PCR result of the *ARSA* 465 site in proband (blue stands for reference gene, and orange stands for *ARSA*).

In Pt19, the mutation c.1160G>T was inherited from his mother and the mutation IVS3+2T>C was a spontaneous splicing mutation. After sequencing the cDNA of *ARSA* from Pt19, it was suggested that this mutation causes exon 3 skipping (Figure 3). Interestingly, mutation IVS3+2T>C could not be detected in Pt19's parents. It indicated that this mutation may be a de novo mutation or that his father was a chimera. Pt14 (c.465G>A) had a compound heterozygous mutation, which has not been reported. His father (c465G>A) had a heterozygous mutation, but his mother had a wild-type allele (Figure 4). The 465 locus was the last base in the second exon of *ARSA*. This synonymous mutation was located between the second exon and second intron. Therefore, we considered that it might affect splicing. Based on the analysis of the child's RNA, we found that after the change of the 465 locus, RNA indeed produced a splicing mutation, which presented as a splicing jump of the 371–465 loci in the second exon (involved 95 bases). In this family, children with a homozygous mutation in the 465 locus might come from the maternal chromosome deletion in this region. Therefore, we performed a fluorescence quantitative PCR for the 465 site and found that the expression of the reference gene in the generation was consistent with the control group. However, the expression of the *ARSA* region was half that of the control group, which confirmed that the proband with the 465 locus had a heterozygous deletion (Figure 5). To determine the deletion region, we designed several primers to amplify the fragment of *ARSA*, including exons 1–3, exons 1–5, and exons 1–8. Interestingly, the proband, father, and mother showed only one band. Thus, we supposed that the proband of the maternal chromosomes contained the entire *ARSA* heterozygous deletion. Therefore, these two mutations (IVS3+2T>C and c.465G>A) are important not only for patient counseling but also for the evaluation of prenatal diagnosis.

Prenatal diagnosis was important for MLD families with *ARSA* mutations. We performed *ARSA* mutation analysis for four MLD families in this study. There was a 25% risk of recurrence and 25% risk of female carriers in all the MLD families with an autosomal recessive genotype. In the Pt5 and Pt15 MLD families, *ARSA* without a mutation provided critical information for parents carrying heterozygous *ARSA* mutations without MLD. This is valuable for prenatal diagnosis in the second pregnancy.

## 5. Conclusions

In summary, 21 Chinese patients were clinically and genetically diagnosed with MLD and analyzed clinically and genetically; four MLD families had additional prenatal diagnoses. MLD is usually caused by the lack of the important enzyme ARSA and results in damage to the nervous system, kidneys, gallbladder, and other organs. In this study, we first found that the patients showed classic clinical symptoms, typical brain MRI findings, and low ARSA enzyme activity of MLD, consistent with previous reports. Furthermore, DNA sequencing detected that all patients carried *ARSA* mutations, including 28 novel and 6 reported mutations. We first reported one coding region mutation that influenced splicing (c.465G>A (p.Lys125ProfsX17)). Prenatal diagnosis was successfully carried out on five fetuses. This study provides more information on critical mutations of *ARSA* in the Chinese population for MLD diagnosis and treatment in the future.

## Figures and Tables

**Table 1:** Summary of major clinical features in 21 Chinese MLD patients.

Pt. ID	Sex	Age onset	Family history	Symptoms at onset	Neurological findings	ARSA enzyme activity	Brain MRI findings
1	Female	5 yrs	−	Psychomotor deterioration, motor regression, mental deterioration	Spastic paraparesis	+	Symmetrical deep WM abnormalities
2	Male	3 yrs	−	Psychomotor deterioration, motor regression	−		Symmetrical deep WM abnormalities
3	Male	6 mon	−	Motor retardation, nystagmus	Nystagmus, peripheral neuropathy		Symmetrical deep WM abnormalities and basal ganglia changes
4	Male	17 mon	−	Psychomotor deterioration, motor regression	Peripheral neuropathy		Symmetrical deep WM abnormalities
5	Male	18 mon	−	Psychomotor deterioration, motor regression	Peripheral neuropathy	+	Symmetrical deep WM abnormalities
6	Female	4 yrs	−	Psychomotor deterioration, motor regression	Dysarthria	+	Symmetrical deep WM abnormalities
7	Male	14 mon	−	Psychomotor deterioration, motor regression, strabismus, nystagmus	Peripheral neuropathy	+	Symmetrical deep WM abnormalities
8	Male	15 mon	−	Psychomotor deterioration, motor regression	Peripheral neuropathy		Symmetrical deep WM abnormalities
9	Female	6 mon	−	Motor retardation	−		Symmetrical deep WM abnormalities
10	Male	16 mon	−	Psychomotor deterioration, motor regression, mental deterioration	Peripheral neuropathy		Symmetrical deep WM abnormalities
11	Female	16 mon	−	Psychomotor deterioration, motor regression	Peripheral neuropathy	+	Symmetrical deep WM abnormalities
12	Female	41 mon	−	Psychomotor deterioration, motor regression	Peripheral neuropathy		Symmetrical deep WM abnormalities
13	Female	7 mon	−	Motor retardation	Peripheral neuropathy	+	Symmetrical deep WM abnormalities
14	Male	18 mon	−	Psychomotor deterioration, motor regression, mental deterioration, strabismus	Peripheral neuropathy	+	Symmetrical deep WM abnormalities
15	Male	27 mon	−	Psychomotor deterioration, motor regression	Peripheral neuropathy	+	Symmetrical deep WM abnormalities
16	Female	28 mon	−	Psychomotor deterioration, motor regression	Peripheral neuropathy	+	Symmetrical deep WM abnormalities
17	Female	12 mon	−	Psychomotor deterioration, motor regression	Peripheral neuropathy	+	Symmetrical deep WM abnormalities
18	Male	12 mon	−	Psychomotor deterioration, motor regression	Peripheral neuropathy	+	Symmetrical deep WM abnormalities
19	Male	15 mon	−	Psychomotor deterioration, motor regression, nystagmus	Nystagmus, peripheral neuropathy	+	Symmetrical deep WM abnormalities
20	Male	12 mon	−	Psychomotor deterioration, motor regression	Dysarthria, peripheral neuropathy	+	Symmetrical deep WM abnormalities
21	Male	15 mon	−	Psychomotor deterioration, motor regression	Peripheral neuropathy	+	Symmetrical deep WM abnormalities

**Table 2:** The *ARSA* genotypes in 21 Chinese MLD patients.

Pt. ID	Mutation	Genetic	Reported	Heritage
1	c.251C>A (pPro84Gln)	Hetero	Novel	Father
1	c.1172T>G (p.Val391Gly)^∗^	Hetero	Novel	Mother
2	c.1172T>G (p.Val391Gly)^∗^	Homo	Novel	Father and mother
3	c.960G>A (p.Trp320Term)	Homo	Novel	Father and mother
4	c.911A>T (p.Lys304Ile)	Hetero	Novel	Father
4	c.1049T>A (p.Leu350Gln)	Hetero	Novel	Mother
5	c.917C>T (P.Thr306Met)	Hetero	Reported	Father
5	c.827C>T (P.Thr276Met)^∗^	Hetero	Reported	Mother
6	c.925G>A (p.Glu309Lys)^∗^	Hetero	Reported	Father
6	c.427T>C (p.Phe143Leu)	Hetero	Novel	Mother
7	c.1130_1132delTCT^∗^	Hetero	Novel	Father
7	c.1238A>G (p.Asp413Gly)	Hetero	Novel	Mother
8	c.244C>T (p.Arg82Trp)	Hetero	Novel	Father
8	c.179_180insCA	Hetero	Reported	Mother
9	c.1130_1132delTCT^∗^	Hetero	Novel	Father
9	c.853C>G (p.Asp283Glu)	Hetero	Novel	Mother
10	c.218C>T (p.Pro73Leu)	Hetero	Novel	Father
10	c.827C>T (P.Thr276Met)^∗^	Hetero	Reported	Mother
11	c.32T>C (p.Leu11Pro)	Hetero	Novel	Mother
11	c.1108-20A>G	Hetero	Novel	Father
12	c.257G>A (p.Arg86Gln)	Hetero	Reported	Mother
12	c.482T>C (p.Leu161Pro)	Hetero	Novel	Father
13	c.925G>A (pGlu309Lys)^∗^	Hetero	Reported	Mother
13	c.302G>T (pGly101Val)	Hetero	Reported	Father
14	c.465G>A (p.Lys125ProfsX17)	Hetero	Novel	Father
14	ARSA del?			Mother
15	c.610C>G (p.Arg204Gly)	Hetero	Novel	Mother
15	c.44G>T (p.Gly15Val)	Hetero	Novel	Father
16	c.640G>A (p. Ala214Thr)	Hetero	Novel	Mother
16	c.893G>T (p.Gly298Val)	Hetero	Novel	Father
17	c.754T>C (p.Ser252Pro)	Hetero	Novel	Father
17	c.1344_1345insCC	Hetero	Novel	Mother
18	c.302_303insG	Hetero	Novel	Father
18	c.1428_1429insC	Hetero	Novel	Mother
19	c.1160G>T (p.387Gly>Val)	Hetero	Novel	Mother
19	IVS3+2T>C	Hetero	Novel	Spontaneous
20	c.830T>C (p.Leu277Pro)	Hetero	Novel	Father
20	c.383T>C (p.Leu128Pro)	Hetero	Novel	Mother
21	c.466G>C (p.Gly156Arg)	Hetero	Novel	Father
21	c.629T>C (pLeu210Pro)	Hetero	Novel	Mother

∗ indicates that mutations were detected more than once in this study.

**Table 3:** The prenatal diagnosis of four families.

Family	Family number	*ARSA* mutation	*ARSA* mutation
Pt5	Proband	c.917C>T (P.Thr306Met) (heterozygous)	c.827C>T (P.Thr276Met) (heterozygous)
	Father	c.917C (wild type)	c.827C>T (P.Thr276Met) (heterozygous)
	Mother	c.917C>T (P.Thr306Met) (heterozygous)	c.827C (wild type)
	Fetus	c.917C (wild type)	c.827C (wild type)

Pt7	Proband	c.1130_1132delTCT (p.Phe377del) (heterozygous)	c.1238A>G (p.Asp413Gly) (heterozygous)
	Father	c.1130_1132TCT (wild type)	c.1238A>G (p.Asp413Gly) (heterozygous)
	Mother	c.1130_1132delTCT (p.Phe377del) (heterozygous)	c.1238A (wild type)
	Fetus 1	c.1130_1132delTCT (p.Phe377del) (heterozygous)	c.1238A>G (p.Asp413Gly) (heterozygous)
	Fetus 2	c.1130_1132TCT (wild type)	c.1238A>G (p.Asp413Gly) (heterozygous)

Pt15	Proband	c.610C>G (p.Arg204Gly) (heterozygous)	c.44G>T (p.Gly15Val) (heterozygous)
	Father	c.610C (wild type)	c.44G>T (p.Gly15Val) (heterozygous)
	Mother	c.610C>G (p.Arg204Gly) (heterozygous)	c.44G (wild type)
	Fetus	c.610C (wild type)	c.44G (wild type)

Pt18	Proband	c.302_303insG (p.L102Pfs) (heterozygous)	c.1428_1429insC (p.S477Qfs) (heterozygous)
	Father	c.302_303insG (p.L102Pfs) (heterozygous)	c.1428_1429CA (wild type)
	Mother	c.302_303GC (wild type)	c.1428_1429insC (p.S477Qfs) (heterozygous)
	Fetus	c.302_303insG (p.L102Pfs) (heterozygous)	c.1428_1429insC (p.S477Qfs) (heterozygous)

**Table 4:** Functional prediction of mutation in *ARSA* with amino acid changing by PolyPhen-2.

Mutation	Function	Score	Sensitivity	Specificity
251C>A (pPro84Gln)	Probably damaging	0.911	0	0.94
1172T>G (p.Val391Gly)	Probably damaging	1	0	1
911A>T (p.Lys304Ile)	Probably damaging	1	0	1
1049T>A (p.Leu350Gln)	Probably damaging	1	0	1
917C>T (P.Thr306Met)	Probably damaging	1	0	1
827C>T (P.Thr276Met)	Probably damaging	1	0	1
925G>A (p.Glu309Lys)	Probably damaging	1	0	1
427T>C (p.Phe143Leu)	Probably damaging	0.999	0.14	0.99
1238A>G (p.Asp413Gly)	Probably damaging	1	0	1
244C>T (p.Arg82Trp)	Probably damaging	1	0	1
853C>G (p.Asp283Glu)	Probably damaging	1	0	1
218C>T (p.Pro73Leu)	Probably damaging	1	0	1
827C>T (P.Thr276Met)	Probably damaging	1	0	1
257G>A (p.Arg86Gln)	Probably damaging	1	0	1
925G>A (pGlu309Lys)	Probably damaging	1	0	1
302G>T (pGly101Val)	Probably damaging	1	0	1
640G>A (p. Ala214Thr)	Probably damaging	0.994	0.69	0.97
893G>T (p.Gly298Val)	Probably damaging	1	0	1
754T>C (p.Ser252Pro)	Probably damaging	0.996	0.55	0.98
1160G>T (p.387 Gly>ValV)	Probably damaging	1	0	1
830T>C (p.Leu277Pro)	Probably damaging	1	0	1
383T>C (p.Leu128Pro)	Probably damaging	1	0	1
466G>C (p.Gly156Arg)	Probably damaging	1	0	1
629T>C (pLeu210Pro)	Probably damaging	1	0	1

## Data Availability

The data related to the article was collected in our clinic.
